# Peripheral nerve ultrasound: a survival guide for the practicing radiologist with updates

**DOI:** 10.1186/s13089-024-00387-0

**Published:** 2025-03-26

**Authors:** Mohamed Ragab Nouh, Hoda Mohamed Abdel-Naby, Tarek El Sakka, Mohamed El-Shafei

**Affiliations:** 1 Department of Radiology, Faculty of Medicine, Alexandria University, 1 Kolyat El-Teb Street Mahta El-Ramel, 2331 Alexandria, Egypt; 2 Present Address: Department of Radiology, Armed Force Hospital, King Abdulaziz Airbase, 31932 Dhahran, Kingdom of Saudi Arabia; 3 Department of Physical Medicine, Faculty of Medicine, Alexandria University, 1 Kolyat El-Teb Street Mahta El-Ramel, Alexandria, 2331 Egypt

**Keywords:** Nerve injury, Neuropathy, Neuro sonography, Neuroma, Ultrasound

## Abstract

Peripheral nerve injuries negatively impact patients’ quality of life and healthcare resources. This review discusses using high-resolution neurosonography (HRNUS) for mapping peripheral nerves and detecting pathologic lesions. It emphasizes the importance of HRNUS in diagnosing nerve disorders and briefs the widely accepted schemes for peripheral nerve injury classification. It also highlights the non-intrusive, flexible, patient-friendly, and cost-effective nature of HRNUS, making it a valuable tool in managing nerve disorders. The authors recommend the use of HRNUS to enable precise diagnoses, prevent permanent disabilities, and contribute to the efficient utilization of healthcare resources.

## Key points

- High-resolution neurosonography (HRNUS) is a feasible and cost-effective tool to map peripheral nerve injuries.
- HRNUS enhances the precision of diagnosing neural and peri-neural lesions.
- A practical guide for radiologists using HRNUS in detecting PN lesions is provided.

## Introduction

Peripheral nerve injury is any damage or trauma to nerves outside the central nervous system. Peripheral nerve (PN) injuries are incapacitating clinical conditions that have deleterious long-term effects on both the patients and the health system resources alike. In today’s patient care guidelines, localizing a peripheral nerve injury is a combined function of clinical neurologic assessment, neurophysiologic studies, and state-of-the-art imaging.

Over the last decades, there have been notable advancements in imaging techniques for detecting and characterizing nerve injuries. While Magnetic Resonance Imaging (MRI) has been the conventional tool for assessing peripheral nerve pathology, especially deep nerves, ultrasound has emerged as the new standard for imaging superficial peripheral nerves. This is due to its non-invasive, dynamic, patient-friendly, and cost-effective nature. However, many radiologists are not familiar with using ultrasound to detect peripheral nerve lesions, leading to the underutilization of this efficient diagnostic tool.

High-resolution neurosonography (HRNUS) can map peripheral nerves, detect pathologic lesions, and conclude a working diagnosis. It has also been effectively used to guide nerve injections and biopsy peri-neural lesions.

This review will briefly overview the generic technical requirements and practical essentials for conducting a sound ultrasound examination of major and common peripheral nerves in radiology practice. Thereafter, we will discuss the necessary anatomical and pathological knowledge required and the common peripheral nerve injury classification schemes used in clinical practice and related to management decisions.

## Peripheral nerve injury epidemiology—the problem

Though peripheral nerve injuries are infrequent, their occurrence has increased over the past few decades, with a prevalence rate of around 2–3% on recent trauma registries [1–3]. Most of these injuries targeted the digital, median, and ulnar nerves in the upper extremity and the planter and peroneal nerves in the lower extremity [1–3]. Interestingly, iatrogenic peripheral nerve injuries account for about a third of these cases [4]. Early recognition of peripheral nerve injuries is paramount for proper patient management, the best possible outcomes, and efficient health system resource management.

## Peripheral nerve imaging—overview

Imaging assessment of peripheral nerves is guided by neurologic assessment and electro-diagnostic (EDx) studies that inform nerve function [5, 6]. The gold standard for imaging neural plexuses and their peripheral territories is magnetic resonance neurography (MRN) [6]. High-resolution neurosonography (HRNUS) offers superior imaging of post-ganglionic superficial peripheral nerve segments when compared to magnetic resonance neurography (MRN) [7, 8]. It has a higher in-line spatial resolution, extended nerve coverage, real-life dynamic assessment of the peripheral nerves, and relatively short scanning time, producing high-definition images. HRNUS also overcomes MRN’s logistical difficulties, such as inaccessibility, limited coil coverage, lack of dynamic assessment, and cumbersome scheduling due to extended exam times [8, 9].

However, the US might sometimes be deemed inapplicable in cases of extensive skin and tissue damage or when intervening gases, bone fragments, or robust metals are present [7]. Further, the use of low-frequency probes may be necessary to penetrate deeply running segments of some peripheral nerves, but this may result in degraded image resolution, and a trade-off has to be considered [7, 9]. Although a previous report [10] pointed to the simplicity of HRNUS after a single-day workshop, becoming skilled in using the US requires a steep learning curve, as the examiner must be familiar with peripheral nerve anatomy, essential sonographic technical requirements and have a sound grasp of variable nerve pathologies [11, 12]. One of the US perks is its ability to manage neurogenic pain by guiding diagnostic and/or therapeutic injections and peripheral neuro-interventions [13, 14]. Although both US and MRI have their unique merits, they are complementary tools. HRNUS can quickly and conveniently scan a whole nerve and its surroundings to direct management, while MRI can corroborate and expand on challenging peripheral nerve lesions, their surrounding interfaces, and supplied muscles.

## HRNUS technical considerations

HRNUS is achievable by most of the current high-frequency (7–18 MHz) linear probes from different vendors. Continued advances in US hardware, dedicated musculoskeletal software presets, and image-processing sophistications resulted in optimized spatial resolution comparable to high-end MR images [8, 9]. Furthermore, the introduction of ultrahigh-frequency (20–70 MHz) linear small transducers, e.g., the hockey stick style, has made superficial digital nerve studies doable [15, 16]. Examining deeply seated nerves, e.g., the sciatic nerve and big body physiques, might require the deployment of lower frequency curvilinear probes (3.5–5 MHz) for adequate US wave penetration and a trade-off between resolution and penetration should be considered [7].

**Fig. 1:**
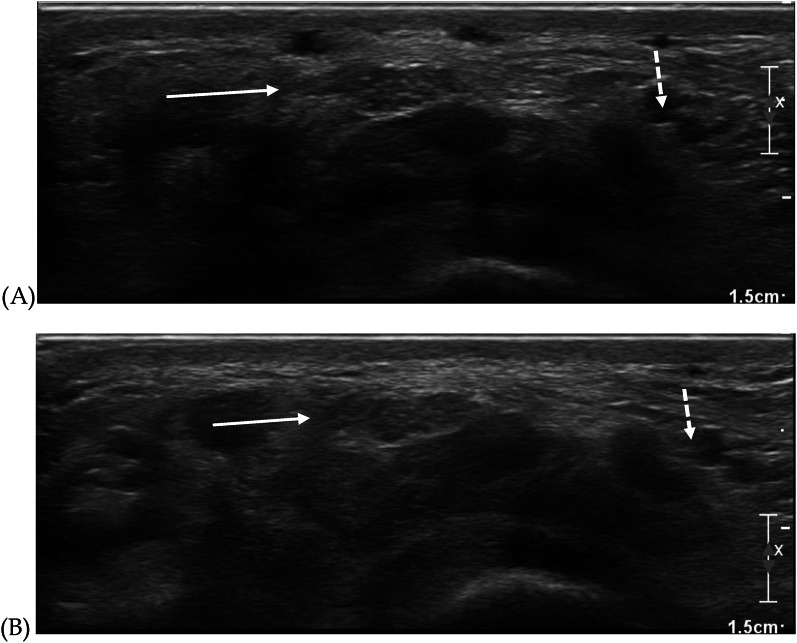
Highlights the crucial role of proper focus adjustment in achieving clear internal neural texture. Axial images **A** and **B** depict the honeycomb pattern of the median (solid arrow) and ulnar (dashed arrow) nerves at different focus levels. **A** Shows the nerves in focus, while **B** has a deeper focus, leading to a loss of the honeycomb appearance due to improper adjustment. The effect is more noticeable in the ulnar nerve due to anisotropy

Optimal examination of peripheral nerves requires proper patient positioning; for both patient and examiner comfort, sufficient gel application, and controlled soft-touch techniques to avoid excessive tissue compression and obtain high-quality diagnostic ultrasound images of peripheral nerves. Furthermore, to obtain an accurate depiction of peripheral nerves, precise depth and focus adjustments are necessary (Fig. 1). This can be achieved by optimizing gain and image zoom settings until the examiner can clearly distinguish the perineurium from the nerve fascicles. Advanced software functions, such as compound and tissue harmonic imaging, along with extended field-of-view panoramic imaging, are essential for enhancing high-resolution HRNUS images [7, 15].

**Fig. 2:**
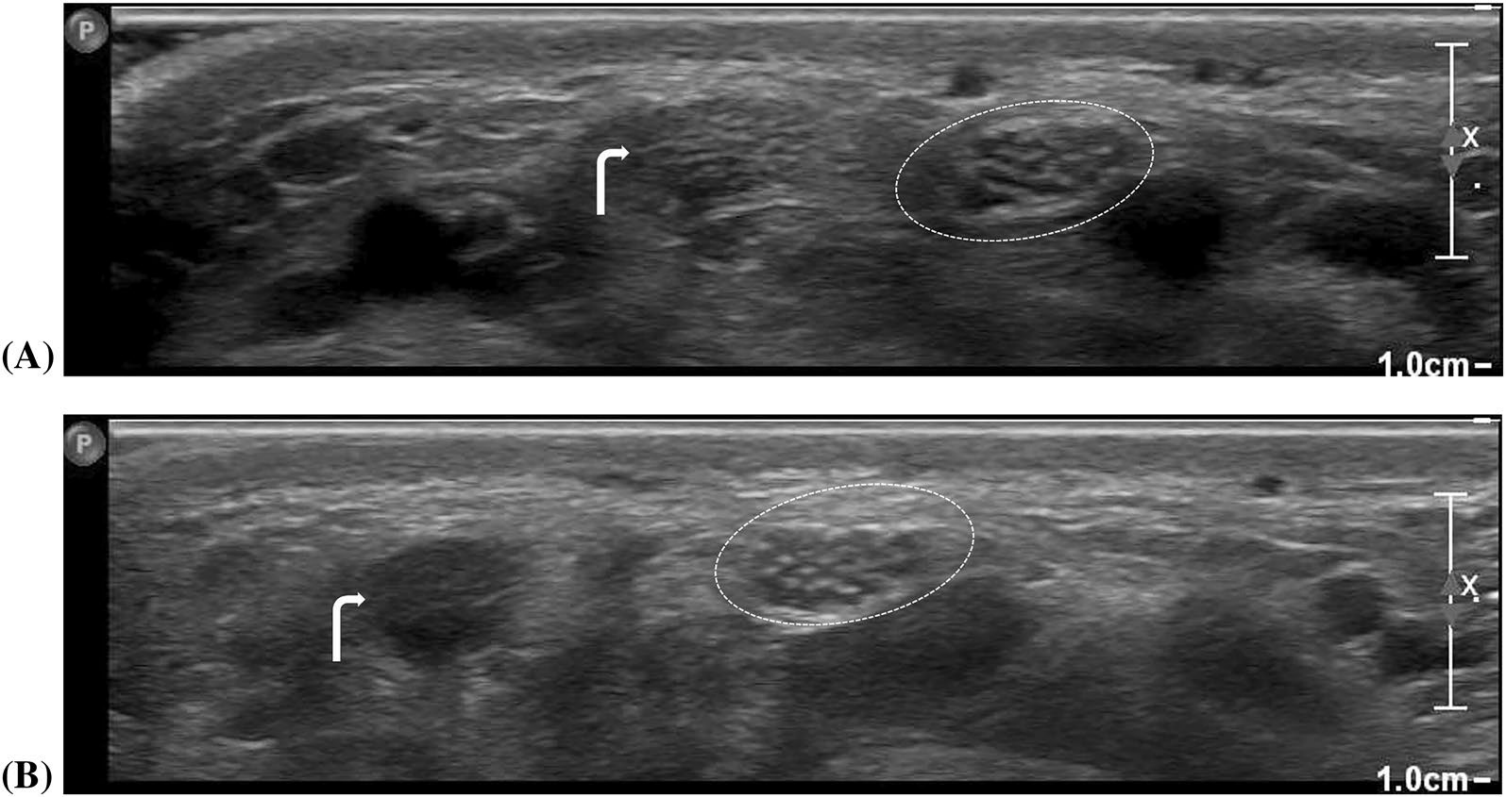
Illustrates the phenomenon of anisotropy, which affects tendons more than nerves. Two images **A** and **B**, of a normal individual’s left-side wrist, showing the median nerve (dashed ellipse) and the adjacent flexor carpi radialis tendon (bent arrows). The images have identical parameters except for probe angulation. The median nerve’s normal fascicular axial appearance is clear in **A** but slightly muffled in **B**. The flexor carpi radialis tendon’s axial appearance is visible in **A** but shadowed in **B** while other tendons appear muffled in both images. M and L refer to medial and lateral directions

**Fig. 3:**
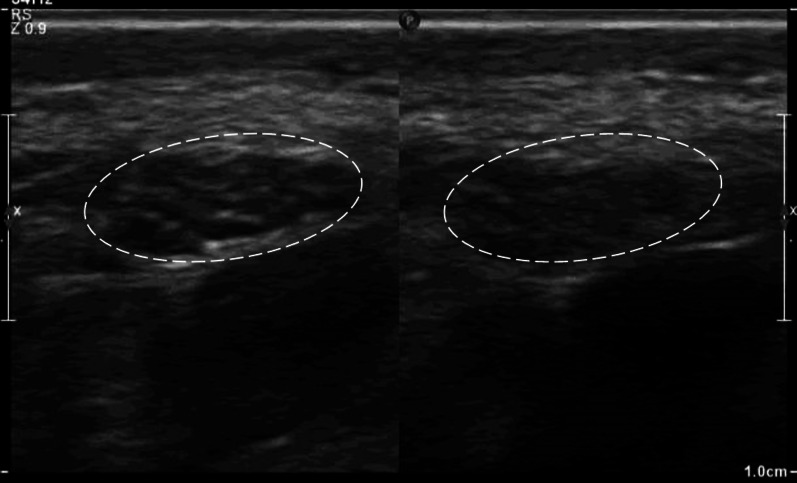
Highlights the importance of considering anisotropy when assessing the pathological state of the median nerve (dashed ellipse). Two axial views of the nerve at the wrist level are shown, with identical parameters except for the probe angulation in the right image. The nerve architecture is visible in the left image but not in the right due to the probe’s angling

**Fig. 4:**
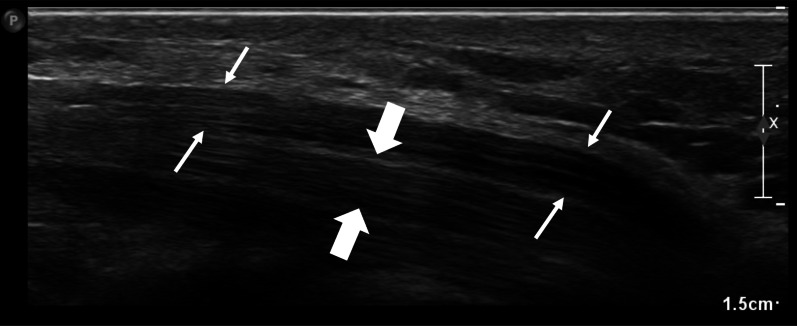
Shows the median nerve (marked between small arrows) and a parallel tendon (marked between block arrows) in the forearm, with the nerve having a tram-track appearance and the tendon having a more compact internal structure. Anisotropy may cause similarity, making differentiation difficult, especially on the left side of the image

Anisotropy (Fig. 2) poses a challenge when imaging highly organized compact structures such as tendons, ligaments, and peripheral nerves. It is a false perceived hypo-echogenicity (Fig. 3) due to the scattering of sound waves reflected back to the transducer caused by subtle probe angulations [17]. To avoid anisotropy and achieve optimal nerve depiction, it is crucial to maintain an orthogonal relationship between the ultrasound probe and the examined part. Nerves exhibit less anisotropy than tendons because of their parallel structure, which gives them smooth surfaces [17] (Fig. 4). Passive limb movement by the examiner during scanning can help clarify confusion when imaging adjacent nerves and tendons.

**Fig. 5:**
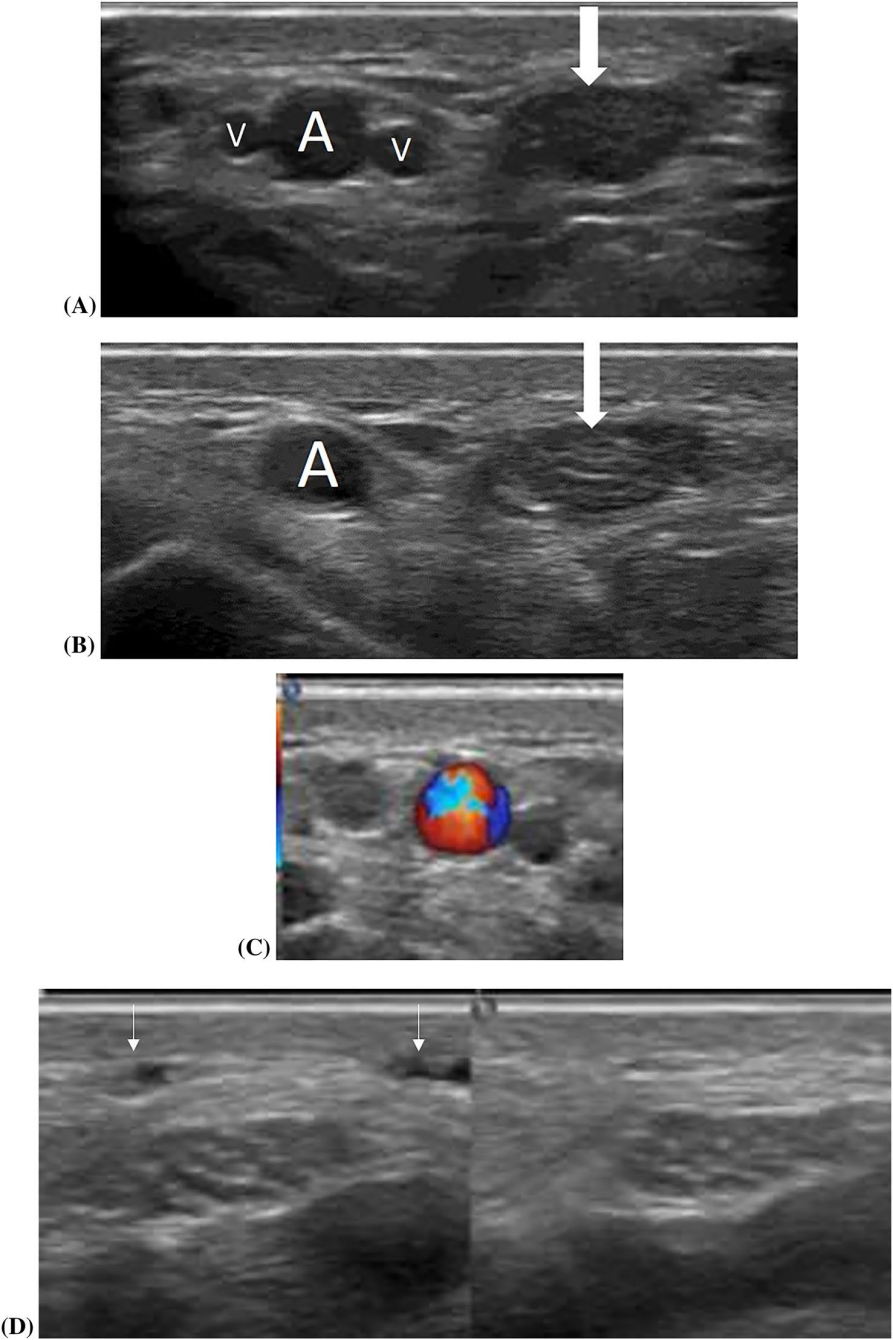
Displays compression and Doppler interrogation abilities to differentiate between arteries, veins, tendons, and nerves. Light probe touch in **A** and gentle compression in **B**, compression reveals total obliteration of the venae comitantes (v), subtle effacement of the radial artery (**A**), and non-deformity of the median nerve (thick arrow) in both **A** and **B**. Color saturation on **C** helps distinguish arteries (asterisk) from nerves and tendons. **D** Subcutaneous veins respond to compression, while the median nerve does not

Doppler imaging, particularly power doppler (PDUS), can help in detecting vessels and distinguishing nerves from accompanying vessels [18]. This discernment is easily achieved through gentle probe pressure, where veins are compressible, arteries are pulsatile and less compressible, and nerves show neither compressibility nor pulsatility [7] (Fig. 5). Addressing the intra- and peri-neural vascularity has been expanded by the recent technologies as Contrast-enhanced ultrasonography (CEUS) and superb micro-vascular imaging (SMI) as will be highlighted in a later section.

Dynamic assessment of peripheral nerve mobility and skeletal muscles is essential for comprehensive peripheral nerve ultrasound exams. This would be elucidated upon under the dynamic sonographic pathologic section.

## Sonoanatomy of the peripheral nerves and innervated skeletal muscles

For a thorough diagnostic sonographic assessment of peripheral nerves, the radiologist must have a comprehensive understanding of the targeted nerve’s anatomy and surrounding structures. Nonetheless, a detailed discussion of individual nerves is beyond the scope of this review. Readers are advised to consult the relevant literature for specific information on a particular nerve.

The peripheral nerves (PNs) are cord-like tracts that form distal to the dorsal root ganglia and span over different body regions to provide a mix of motor and sensory functions. PNs reach their destinations by traveling tortuous courses through fat and/or fascial planes wrapping muscles [7, 19, 20].

Morphologically, the basic structural units of peripheral nerves are the fascicles that consist of a group of bundled nerve axons and supporting mesenchyme; the perineurium, wrapped in and compartmentalized by a layer of connective tissue called the epineurium. Their ultrasound imaging features are reflections of this structured organization where peripheral nerves appear as hypoechoic fascicles defined by hyperechoic epineurium and interfascicular perineurium, displaying a distinctive honeycomb (Fig. 2) and tram-track appearance (Fig. 3) on both transverse and longitudinal US scans, respectively and distinguishing them from adjacent tendons [7, 19, 20].

**Fig. 6:**
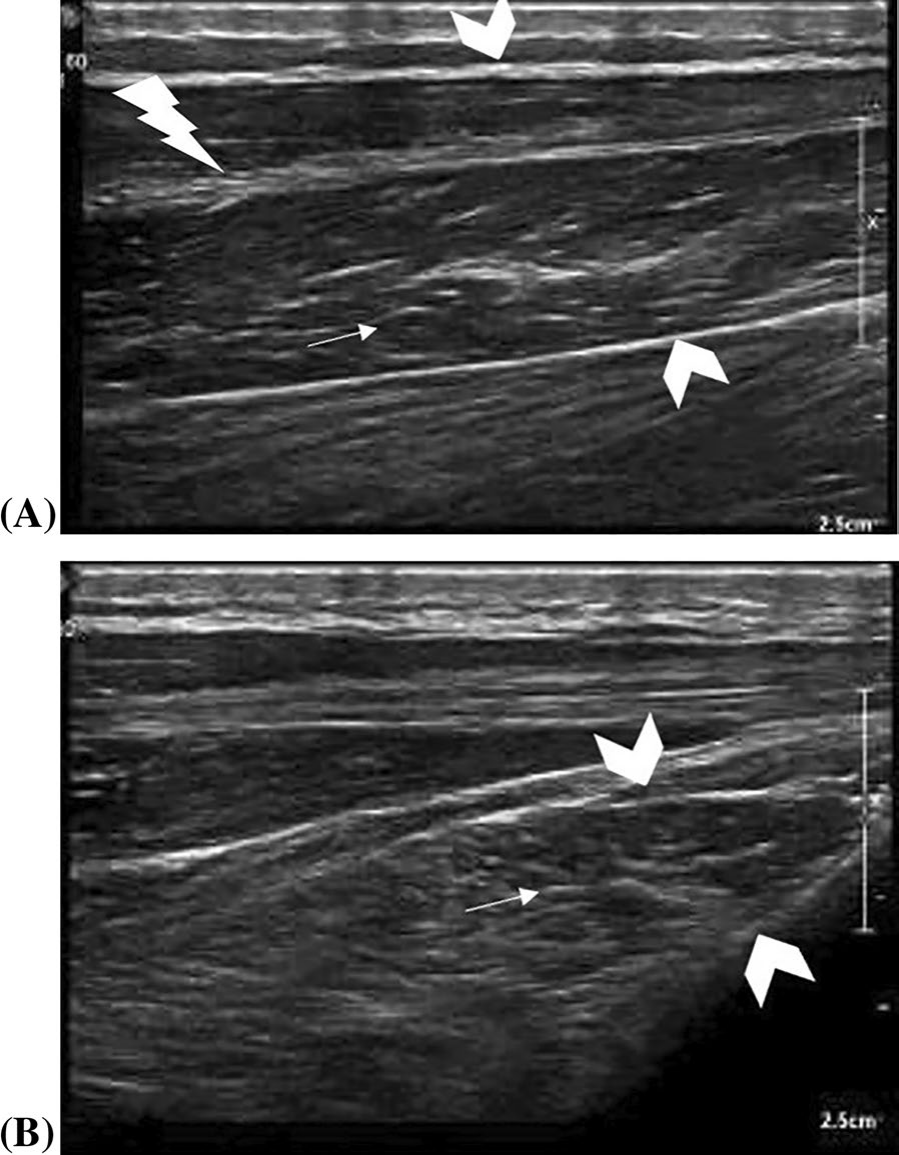
Shows normal skeletal muscle, the soleus, sonographic features. The muscle fibers appear scattered and hypoechoic, enveloped and intertwined by thin echogenic structures of the epi-(thick chevron) and peri-(small arrow) mysium. These arrangements give the muscles a feathery appearance on the longitudinal plane (**A**) and a mottled appearance on the axial plane (**B**). Note the central tendon (lightning Bolt) due to the bi-pennate type of soleus muscle

For a thorough RNUS exam, it is crucial to evaluate the skeletal muscles supplied by the peripheral nerve being examined. Healthy skeletal muscles appear diffusely hypoechogenic, due to well-organized muscle fibers with high water content, surrounded and interwoven by thin linear echogenic epi- and peri-mysium giving muscles their characteristic feathery and mottled appearance on longitudinal and transverse ultrasound scanning, respectively [21] (Fig. 6).

## Nerve trauma pathophysiology essentials

### Mechanisms

Peripheral nerves are vulnerable to a wide range of traumatic events due to their long courses, articular crossings, and occasional point fixations along their way to their targeted effectors. Various mechanisms can impact the peripheral nerves either alone or in combination, such as compression, stretching, crushing, laceration, or complete severance, disrupting the nerves’ physiological balance and/or anatomical integrity [7, 9]. Moreover, peripheral nerves are susceptible to injuries caused by various factors such as daily traumatic events like occupational stress, household chores, road traffic accidents, and field combats, or due to medical procedures like articular reductions, arthroscopy, etc. Repetitive micro-trauma, as seen in different entrapment syndromes, is also a significant cause of nerve damage [1–4, 7].

### Pathophysiology of peripheral nerve injury

Functionally, axons and their sheaths are the stress-bearing unit of the PNs. Peripheral nerve injury induces the release of different signaling stimuli and local chemo-mediators that alter endoneural vascularity, myelin diffusivity, and homeostasis with subsequent affection of nerve fibers [22, 23]. Accordingly, minor forces in the acute traumatic events or early compression may just cause neural edema along with variable degrees of neural transmission disruption with preserved gross nerve integrity (comparable to neuropraxia). Nonetheless, with escalating applied forces, variable degrees of disruption involve the individual nerve fibers and their surrounding connective tissues (comparable to neurotmesis and axonotmesis). While the distal segments of the cut axons will undergo Wallerian degeneration, the proximal segments will sprout in trial to reunite to their abandoned distal segments guided by the surrounding connective tissue components that act as a guiderail. Aberrations of this regenerative process may occur, and sprouting neurons inadvertently fuse with proliferating perineural connective tissues, producing localized neuro-mesenchymal aggregate called neuroma, which may connect the opposing ends of the injured nerve as a focal bump named a neuroma-in-continuity or crown the severed end; named an end-stump neuroma [24, 25].

Seddon [26] classified nerve damage into three distinct pathological categories: neurapraxia, axonotmesis, and neurotmesis. Neurapraxia represents the mildest form of nerve damage and involves interruption of microscopic axonal myelin transfer (demyelination) without gross disruptions. Axonotmesis, on the other hand, results in injury to the axon, while the nerve sheath remains intact. Neurotmesis, the most severe form, is characterized by complete nerve severance. Sunderland [27] further expanded axonotmesis depending on the extent of surrounding connective tissue damage to become five tires category and Mackinnon [28] added a sixth category (Grade VI) for mixed injuries. A clear understanding of these categories can aid in the accurate diagnosis and treatment of nerve damage.

### Classification of nerve injuries

The Seddon [26] and Sunderland [27] classifications are two widely implemented clinicopathological grading systems used to describe and manage peripheral nerve injuries (Table 1). Overall, Sunderland I–III are lesions of minimal micro-structural disruptions that are commonly managed conservatively via active surveillance, while Sunderland IV and V are major nerve injuries that necessitate timely surgical intervention for the best management outcomes.

**Table 1:** Summary of the peripheral nerve injury working classifications, pathophysiology, sonographic findings, and management perspectives

Seddon classification	Sunderland classification	Pathophysiology	NUS findings	Clinical imaging/perspective	Management perspective
Neuropraxia	Grade-I	Disrupted myelin	Focal hypoechoic thickening	Low-grade nerve injury	Non-surgical
Axonotmesis	Grade-II	Disrupted axon			
	Grade-III	Disrupted endoneurium	Focal hypoechoic thickening ± nearby S.C. scar		
	Grade-IV	Disrupted fascicle, epineurium, and perineurium	Spindle neuroma epineural scar encasement	High-grade nerve injury	Surgical
Neurotmesis	Grade-V	Complete nerve severance	Stump neuroma		

## Scanning technique and landmarks for peripheral nerve imaging

To scan a peripheral nerve, it is sensible to first locate it at a common anatomical landmark easily accessible to the ultrasound (Table 2). Additionally, peripheral nerves accompany vessels along their paths, which helps in their identification. They can be clearly distinguished from their surroundings by their characteristic sonographic neural print. After that, the nerve can be traced up and down in a trail-like pattern along its expected course both axially and longitudinally. It’s essential to scan the entire course of the nerve to identify the type of injury, the gap distance, and any neuromas or associated perilesional fibrotic scar not defined by EDx studies. This approach has proven to be highly credible and reproducible in NUS [7, 29].

**Table 2:** Summarized common anatomic landmarks of the upper and lower extremities major peripheral nerves along their courses

Extremity	Nerve in question	Anatomic region	Common landmarks
Upper limb	Brachial plexus	Supraclavicular region	The anterior and middle scalene muscles and the proximal subclavian artery for the brachial plexus
		Upper arm	The brachial artery
		Forearm	The flexor digitorum superficialis and profundus muscles in the forearm
	Median	Wrist	The carpal tunnel
	Ulnar	Wrist	The ulnar artery at the wrist
		Elbow	The medial epicondyle at the elbow
	Radial	Arm	The radial groove in the arm
Lower limb	Sciatic	Gluteal region	Ischial tuberosity and hamstring origin for the proximal sciatic nerve
		Popliteal fossa	The popliteal artery for the distal sciatic nerve
	Posterior tibial	Knee (posteriorly)	The medial and lateral heads of the gastrocnemius in the proximal calf
		Leg	The posterior tibial artery in the middle and distal calf
		Ankle	The medial malleolus at the ankle
	Peroneal (fibular)	Knee	The fibular head, below knee
	Medial plantar	Plantar foot	Parallel to the MPA, between the FHB and QP muscles

*MPA* medial plantar artery, *FHB* flexor hallucis brevis muscle, *QP* quadratus plantae muscle

## HRNUS pathologic findings

HRNUS can help locate and assess the magnitude of confounding PN, complementing physical examination and EDx studies [7, 29]. Peripheral nerve pathologies can cause morphological changes such as abnormal caliber, altered architecture or contour, or discontinuity [7, 29]. Furthermore, dynamic assessment of nerve mobility and surrounding tissues, including innervated muscles, is critical for a comprehensive ultrasound examination [19, 20].

In mild injuries, the role of ultrasound may be caveated by absent morphologic changes. However, it is essential to confirm nerve integrity and identify co-morbidities that may hinder nerve recovery, e.g., nearby collections or foreign bodies, warranting early intervention to alleviate localized pressure and promote sound peripheral nerve recovery [7, 29].

### Sonographic findings of peripheral nerve disease

A nerve injury can result in various morphological changes, including nerve swelling or flattening, loss of nerve bundle continuity, neuroma formation, and scar tissue in and/or around the nerve. Additionally, there may also be neural structural changes, such as altered echogenicity due to spatial heterogeneity of its alternating hypo- and hyperechoic linear components.

#### Morphologic changes

Recognizing subtle alterations in a peripheral nerve segment that has been impacted by mild neuropathy can pose a challenge, particularly in cases of trauma. Ultrasound imaging may not be capable to distinguish between neurotmesis and high-grade axonotmesis without physically separated nerve endings as a neuroma in continuity may form in either case. Therefore, Cross-checking the comparable contralateral nerve and/or adjacent regional nerves is an advisable golden rule to document these changes whenever suspected [30].

**Fig. 7:**
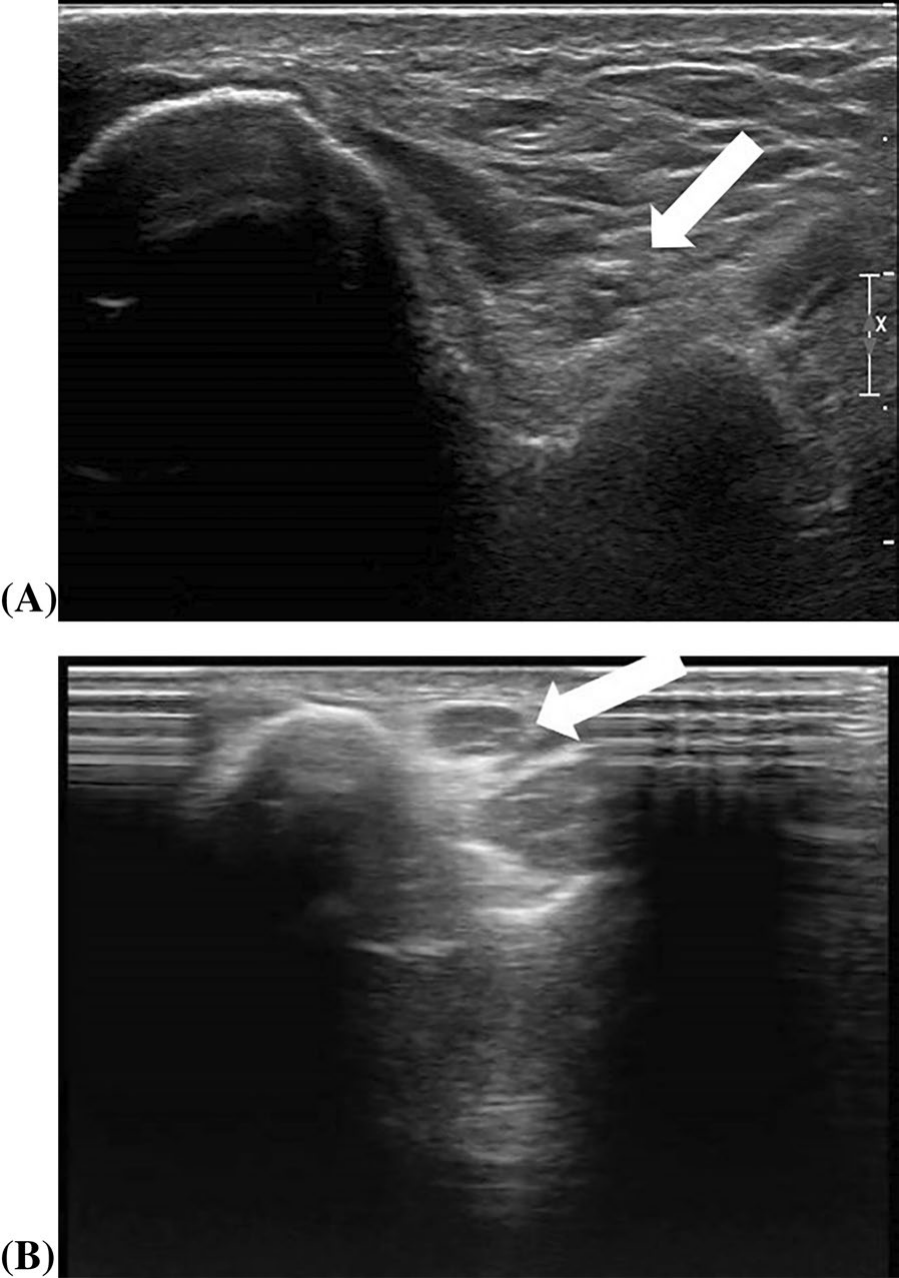
Compares the position of the ulnar nerve (arrow). In **A**, the nerve is in its normal position, the retro-condylar groove behind the medial humeral epicondyle, while in **B**, it is abnormally mobile and located on the inner edge of the medial epicondyle and blurred with widened hypo echogenicity between the echogenic fasciculi

Morphological changes of nerves on HRNUS can manifest in various ways, such as (1) Enlargement: nerve may be swollen and hypoechoic due to intra-neural edema (Fig. 7), or focal swelling from intra-neural ganglion formation [31, 32], (2) Atrophy: this is commonly seen in compressive neuropathies with flattening of the compressed segments, and edema-induced enlargement of the prior segments, (3) Nerve discontinuity: peripheral nerve defects may be classified as complete or incomplete based on axonal fiber tear extent. (4) Contour irregularities: especially in compressive neuropathies and remote closed injuries as by tractional forces that eventually scar the perineurium or surrounding fascial planes and may challenge the ultrasound discernment of nerve fascicles. At times, this may require the use of MRI imaging for further clarification [7, 9]. (5) Calcifications: HRNUS has successfully identified linear calcifications in the median and ulnar nerves, revealing neuritis ossificans confirming a neural rather than a vascular nature [33, 34].

**Fig. 8:**
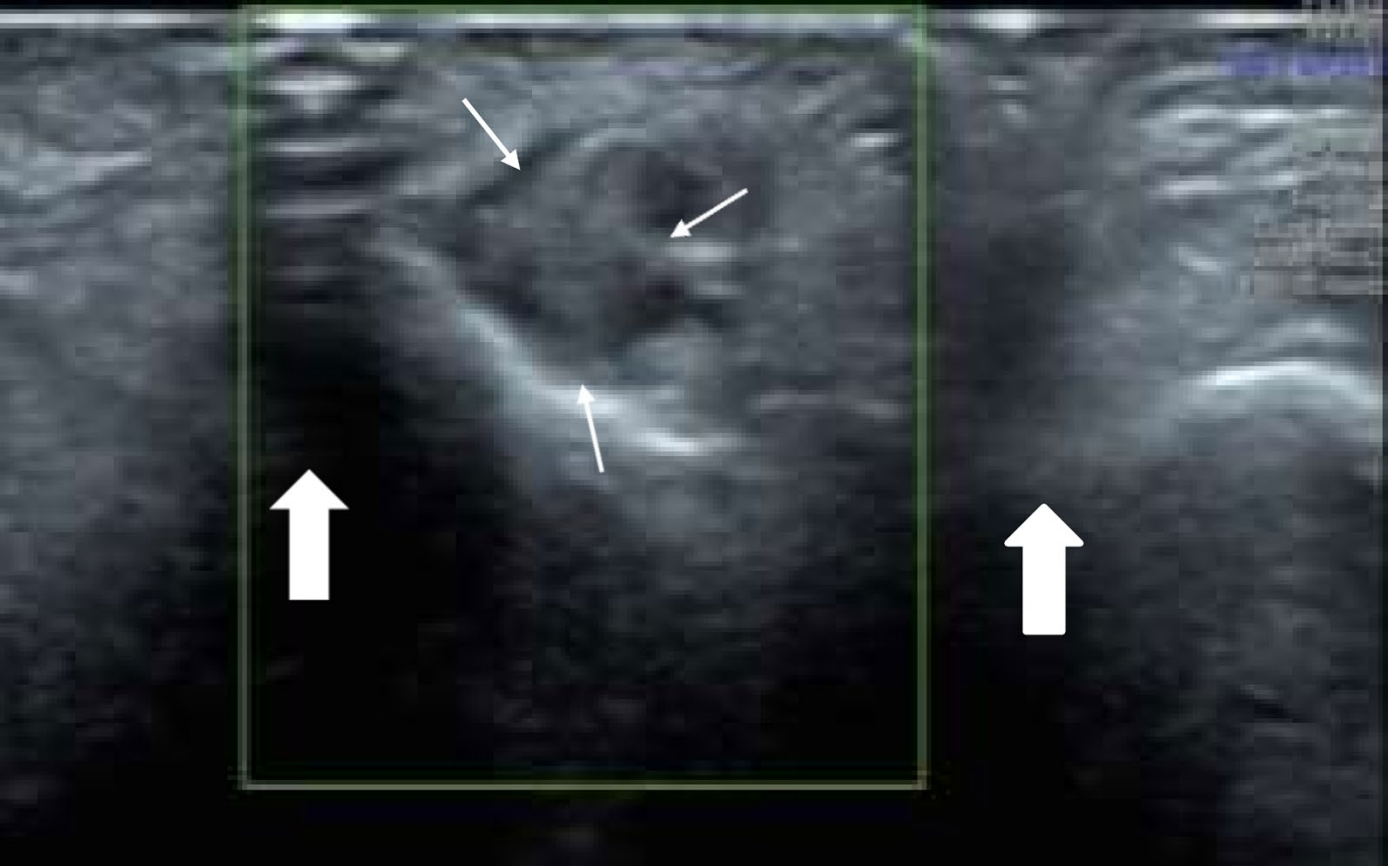
Shows Morton’s neuroma (small arrows) in the third inter-metatarsal space on the plantar aspect of the toes in the axial plane through a plantar approach. The neuroma appears as a local enlargement of the nerve of varying hetero-echogenicity. Note that the artifacts (block arrows) are due to loss of contact between the probe and skin, which can be prevented by adding a thick gel pad in-between

One crucial finding in morphologic HRNUS that warrants special attention is the formation of neuroma, a focal reparative growth that often follows nerve damage. It can occur after amputations, remote traction and lacerative trauma, or in compressive neuropathies [7, 8, 35, 36]. Neuromas can vary greatly in size, and larger neuromas are associated with a negative prognosis [37, 38]. Sonographically, neuromas are characterized by local enlargement of the nerve of varying hetero-echogenicity due to intermingled haphazardly oriented neuro-mesenchymal tissue components (Fig. 8). This might be recognized as a focal nodular, expansion of the nerve contour in case of neuroma in continuity or as a localized swelling at the end of a transected nerve in case of an end-stump neuroma [7, 8, 35, 36]. Besides, confirmation of their presence can be achieved through sonopalpation, which may trigger paresthesia in the sensory nerve distribution known as the sonographic Tinel’s sign [39]. It is imperative to give diligent consideration to the identification of neuroma on HRNUS, as it is critical for management decisions [38].

Although visual gestalt of morphological changes in peripheral nerves is common in clinical settings, there been an increasing interest in using quantitative metrics such as cross sectional area, texture analysis and fascicular density to establish a standardized assessment of nerve damage. This approach can facilitate monitoring of the healing process, reduce the need for watchful expectancy periods; in cases where the regenerative potential is unknown, and enable early surgical intervention for better outcomes [40–43].

The cross-sectional area (CSA) is a reliable quantitative biometric index for nerve abnormality in different peripheral neuropathies, notably compressive neuropathies affecting the upper and lower extremities [44–48]. There is a growing interest in establishing normative data reference values for peripheral nerve measurements in various ethnic groups as it varies with ethnicity, age, and the body physique [49–51]. It is noteworthy that CSA measurements ought to exclude the outer epineurium, which can reactively thicken due to repetitive micro-trauma, such as nerve subluxation [30, 44].

Hence, HRNUS can distinguish between partial fascicular disruptions and complete severance, recognize nerve gapping, and identify neuromas for proper surgical planning which necessitates accurate identification of nerve damage, when clinical and EDx studies were predicaments [35, 38]. Furthermore, it has been successfully employed in peripheral neuroma management by guiding pharmacologic nerve blocks and radiofrequency ablative procedures [13, 52].

#### Structural alterations—echogenicity and/or architecture

On HRNUS, effacement of the normal neural ultrasound print could be an early indicator of peripheral nerve irritation. This is perceived as widening of intra-neural fascicles hypo-echogenicity, and loss of ordered fascicular echotexture of a nerve segment due to intraneural venous congestion and edema [7, 9, 15, 53].

Damage to the nerve perineurium or surrounding connective tissues can result in perineural fibrosis and tissue scarring, which can be identified as focal neural and peri-neural irregularities with altered contour and echogenicity [7–9, 36]. Scars are challenging to visualize using ultrasound due to their perpendicular nature, resulting in vaguely defined hypoechogenic longitudinal lines in skin, subcutaneous tissue, and fascia interrupting the normally smooth architectural features of these tissues [54]. Significant epineural fibrosis may predict the need for surgical or interventional neurolysis in the management of peripheral neuropathy [38].

#### Doppler assessment

Normal peripheral nerves should not show blood flow during clinical color Doppler exams [7, 18, 55]. Power Doppler is better at detecting intra and peri-neural vascularity in compressive neuropathies [55, 56]. Superb microvascular imaging (SMI), which will be highlighted in a later section, has expanded sonographic capabilities, allowing for limitless depiction of vascular flow [57]. Therefore, Deeg et al. [58] recently reported that the absence of visible intraneural vasculature as a negative finding in the diagnosis of compression neuropathies should be interpreted with caution, as the intraneural vascularity may be depicted beyond the 18 MHz resolution power of a transducer.

While there is currently no reported evidence of altered intra-neural vascularity resulting from acute nerve trauma, doppler studies have demonstrated their usefulness in evaluating compressive neuropathic syndromes in both the upper and lower limbs [59, 60]. It can demonstrate adjacent vascular anomalies that are associated with compressive neuropathies, such as a bifid median nerve and persistent median artery [61]. Additionally, Doppler evaluation may reveal unexpected findings that could cause peripheral neuropathy in certain clinical situations, such as a compressive aneurysm [62].

## Dynamic assessment of peripheral nerve and perineural tissue by HRNUS

Peripheral nerves are dynamic structures that can adapt to mechanical stresses caused by changes in position, particularly over joints [63]. Neurodynamic testing is typically conducted alongside peripheral nerve ultrasound examinations to assess these dynamics when clinically applicable, a perk for ultrasound over MRI [8, 9].

HRNUS demonstrated varying peripheral nerve excursions in both healthy and symptomatic individuals [63–65]. Nonetheless, caution must be exercised when evaluating this criterion as it may have an impact on detrimental biometric indices, such as CSA in normal asymptomatic subjects, and different joint positions, which require interpretation in the appropriate clinical context [63, 66, 67].

Hyper-mobility has been theorized as a risk factor for peripheral neuropathy, attributed to neural edema and peri-neural fibrosis from repeated friction on peripheral nerves and peri-neural interfaces [66] (Fig. 6). HRNUS has reported reduced median nerve excursions and deformability in patients with carpal tunnel syndrome compared to normal controls during varied wrist and finger movements [67, 68].

At times atypical neurogenic pain can happen sequel to local irritations and subclinical trauma, especially in the upper extremities [69]. Ultrasound imaging has shown a characteristic hourglass-shaped appearance in such cases [69, 70]. This may be due to localized neural and epineural edema creating fixation points for nerve fascicles, ultimately resulting in twisting of nerve fascicle or nerve torsion; commonly described in the radial nerve [69]. Early surgical intervention is critical to preserve neural functionality, and it is therefore essential to recognize the condition promptly [69, 70]. Also, dynamic ultrasound examination may reveal adhesions and restricted movement of the nerve and its surroundings e.g. tendons, due to scarring in the traumatic settings, as described previously [7, 71].

## Ultrasound of the perineural spatial environment

HRNUS aids in the assessment of the perineural extrinsic environment to disclose potential confounding factors that compromise the nerve or potentially hinder its recovery, such as accessory muscles and/or ossicles [72–74], ganglia [75], foreign bodies [38], hormonal implants [76], bony fragments [38], surrounding collections [77, 78], scar tissue [71], and adjacent aneurysmal compression [71]. Even more, the US has assisted in retrieving noxious factors resulting in neuropathy [76, 79]. Thus, HRNUS has the ability to differentiate between peripheral nerves that require urgent exploration and those that can be treated conservatively.

## Supplied skeletal muscles sonographic findings

Occasionally, muscular changes may be the only indicator of a remote injury of the more proximally supplying nerve. This is especially notable following tractional trauma with subsequent severance of a nerve at a fixation point along its course as in musculocutaneous nerve severance between biceps and brachialis in brachial plexus injuries. In this sense, no peripheral ultrasound exam would be considered comprehensive without assessment of the supplied muscular territories of the examined nerve. Nonetheless, muscle denervation changes are subtle and non-specific in the acute/subacute phase of nerve injury compromising ultrasound validity during these times [80]. However, in the delayed stages reduced size of the muscles along with their increased echogenicity are well recognized features on ultrasound [81].

Following injury of a motor nerve, denervation changes commences in the supplied muscle(s). Early in the stage of denervation edema, US will not exhibit specific morphologic changes [7, 21]. Later on, relative reduced muscle volume and variable increased echogenicity is recognized when compared to the adjacent muscle groups and/or the corresponding contra-lateral encounter(s). Finally, when fatty atrophy ensues the muscle would be globally hyperechoic on ultrasound [7, 21].

Muscular changes might be the sole indicator of a remote injury of the more proximally supplying nerve; especially after tractional trauma subsequent severance of a nerve at a fixation point along its course. In this sense, no peripheral ultrasound exam would be comprehensive without assessment of the muscular territories of the examined nerve [7, 21].

Muscle denervation changes are subtle and non-specific in acute/subacute nerve injury where denervation muscle edema initially lacks specific morphologic changes, compromising ultrasound validity in these phases. Nonetheless, in delayed stages, reduced muscle size and increased echogenicity are recognized features on ultrasound [80, 81].

## Spotlights on peripheral nerves ultrasound advancements

There is a growing interest in adopting quantifiable metrics to enhance the reliability of HRNUS results and minimize inter-rater variations due to operator dependence. Furthermore, limitations of color and power doppler in evaluating peripheral neuropathies have prompted the search for a more powerful tool to assess intra- and peri-neural vascularity.

### Quantitative ultrasound (QUS)

Quantitative ultrasound (QUS) techniques have been introduced to provide a more objective evaluation of peripheral nerves, optimize the yield of high-resolution nerve ultrasound, and reduce the operator and system assessment biases.

Some of these have been adopted in clinical settings, such as B-mode echogenicity measurements and elastography (both strain and shear-wave), while others, such as the characterization of raw backscattered ultrasound radiofrequency signals, are still being investigated in laboratory trials.

Several B-mode echogenicity metrics have been studied in both clinical and cadaveric models such as US nerve density [82], nerve hypoechoic fraction and cross-sectional area (CSA) [42], nerve–tissue contrast index (NTI) [43], and gray level co-occurrence matrix (GLCM) [40].

### Portraying peripheral nerve microcirculation

The link between altered peripheral nerve vascularity and peripheral neuropathies as well as peripheral nerve regeneration has been demonstrated [83, 84]. Therefore, the goal of research is to portray peripheral nerve microvascularity in order to develop reliable clinical surveillance tools.

Contrast-enhanced ultrasonography (CEUS) outperforms power doppler thanks to its superior signal-to-noise ratio and dynamic intravascular distribution. It demonstrated superior efficacy in identifying intra- and peri-neural microvascular patterns of peripheral nerves in animal models [85, 86]. Nonetheless, its applicability in human studies is currently underexplored and limited to carpal tunnel syndrome [87, 88].

Another advancement is the development of super-resolution ultrasound which uses contrast microbubbles to detect micro-vessels and provide micron-scale spatial resolution at clinically relevant depths. It is an ultrafast imaging tool that is capable of resolving high-frame rate images by localizing microbubbles and deblurs it to produce super-resolved vascular images with higher spatial resolution. However, it is still limited to laboratory and animal trials and underused in the clinical settings for its limitations [89].

To overcome the inherent invasiveness of CEUS among other limitations, superb micro-vascular imaging (SMI) has been devised. SMI is an advanced ultrasound technique that visualizes low-velocity blood flow in small vessels by analyzing tissue movements and removing clutter signals to reduce motion artifacts mapping only signal from intra-vascular volume. It outperforms color and power doppler techniques in terms of micro-vessels imaging [90, 91]. However, there is currently only limited literature, with the main focus on carpal tunnel syndrome [92].

These techniques enhance peripheral nerve vascular imaging and is going to provide valuable insights into these microvascular structures in clinical settings.

### Ultrasound elastography of peripheral nerves

US elastography tests tissue elasticity by analyzing the tissue’s response to an applied force [93]. Elastography techniques have been deployed for objective evaluation of normal peripheral nerves stiffness [94, 95], the early diagnosis of diabetic peripheral neuropathy (DPN) [96, 97] and compressive neuropathies as carpal tunnel syndrome [98, 99] as well as other peripheral nerve disorders [100].

## Summary

High-resolution nerve ultrasonography (HRNUS) is a ubiquitous imaging modality for evaluating peripheral nerve pathologies, particularly traumatic injuries. It is indispensable for general radiologists to learn, absorb and master HRNU as it provides precise identification, delineation, and triage of suspected nerve lesions, equipping physicians with vital information for tailored management strategies. HRNUS is especially beneficial for healthcare systems with limited resources, enabling the expedite identification of patients in need of immediate medical attention for a speedy recovery.

## Data Availability

Not applicable.
